# Metabolite profiling and associated gene expression reveal two metabolic shifts during the seed-to-seedling transition in *Arabidopsis thaliana*

**DOI:** 10.1007/s11103-017-0665-x

**Published:** 2017-10-18

**Authors:** Anderson Tadeu Silva, Wilco Ligterink, Henk W. M. Hilhorst

**Affiliations:** 1 Laboratory of Plant Physiology, Wageningen University, Droevendaalsesteeg 1, 6708 PB Wageningen, The Netherlands; 2 Present Address: Interdisciplinary Plant Group, University of Missouri, Columbia, MO USA

**Keywords:** Metabolite–metabolite correlation, Metabolite profile, Metabolite-transcription correlation, Network analysis, Seedling establishment, Seed-to-seedling metabolism

## Abstract

**Key message:**

Metabolic and transcriptomic correlation analysis identified two distinctive profiles involved in the metabolic preparation for seed germination and seedling establishment, respectively. Transcripts were identified that may control metabolic fluxes.

**Abstract:**

The transition from a quiescent metabolic state (dry seed) to the active state of a vigorous seedling is crucial in the plant’s life cycle. We analysed this complex physiological trait by measuring the changes in primary metabolism that occur during the transition in order to determine which metabolic networks are operational. The transition involves several developmental stages from seed germination to seedling establishment, i.e. between imbibition of the mature dry seed and opening of the cotyledons, the final stage of seedling establishment. We hypothesized that the advancement of growth is associated with certain signature metabolite profiles. Metabolite–metabolite correlation analysis underlined two specific profiles which appear to be involved in the metabolic preparation for seed germination and efficient seedling establishment, respectively. Metabolite profiles were also compared to transcript profiles and although transcriptional changes did not always equate to a proportional metabolic response, in depth correlation analysis identified several transcripts that may directly influence the flux through metabolic pathways during the seed-to-seedling transition. This correlation analysis also pinpointed metabolic pathways which are significant for the seed-to-seedling transition, and metabolite contents that appeared to be controlled directly by transcript abundance. This global view of the transcriptional and metabolic changes during the seed-to-seedling transition in Arabidopsis opens up new perspectives for understanding the complex regulatory mechanism underlying this transition.

**Electronic supplementary material:**

The online version of this article (doi:10.1007/s11103-017-0665-x) contains supplementary material, which is available to authorized users.

## Introduction

Seed germination is a critical stage in the plant’s life cycle which starts with the uptake of water by the dry seed, followed by embryo expansion and (commonly) radicle emergence. This process is characterized by a transition from a quiescent- to a metabolically highly active state (Penfield et al. 2005; Fait et al. 2006). Uptake of water by seeds is tri-phasic with a rapid initial uptake (phase I, *imbibition*). Phase I of seed germination occurs without visible morphological changes, and is characterized by the initiation of seed-specific germination metabolism that prepares the seed for radicle protrusion (Fait et al. 2006). Phase I is followed by a plateau phase (phase II) in which water content is constant but metabolic activity increases. Radicle protrusion through the embryo-surrounding structures marks the end of phase II and a further increase in water uptake (phase III) occurs as the embryonic axis elongates and the embryo establishes itself as a young seedling (Bewley et al. 2013).

Arabidopsis mutants have been extensively used for the functional analysis of genes involved in seed germination (Debeaujon and Koornneef 2000; Lu and Hills 2002; Fulda et al. 2004; Yang et al. 2013). Some mutations slow down seed germination but do not significantly arrest it. This phenomenon may occur as a result of a significant reduction in oil reserve content accumulated during seed maturation (Focks and Benning 1998; Lu and Hills 2002; Penfield et al. 2005). However, another study with a mutant deficient in plastidic pyruvate kinase (*pkp1*) suggested that delayed seed germination and, consequently, seedling establishment is not caused specifically by a lack of seed oil reserves but may be related to reduced pyruvate kinase activity during germination (Andre and Benning 2007). These observations suggest that seedling establishment is not only affected by mobilisation of reserves accumulated during seed development, but also by additional metabolic processes across developmental stages during the seed-to-seedling transition. Phase transitions such as that from seed to seedling require the integration of metabolite and transcript status. It has been shown that elevated sucrose or glucose levels are associated with seedling establishment (Borisjuk et al. 1998, 2002). However, other metabolic processes also take place at high carbohydrate contents, and these may also play an important role in seedling establishment (Tognetti et al. 2013). In addition, sugar pathways have been shown to be in cross-talk with nitrogen pathways (Coruzzi and Zhou 2001).

It is known that amino acids are the major transport form of N in plants (Tegeder and Ward 2012; Tegeder 2014) and growth is dependent on N supply, assimilation and utilization (Stitt and Krapp 1999). The C/N balance is decisive for the regulation of gene expression by carbohydrates and nitrogen (Zheng 2009). Likely, metabolic regulation of the heterotrophic to autotrophic transition extends beyond primary metabolism. However, it is possible to identify primary metabolites involved in gene expression events, by examining their pattern in relation with the expression of specific genes (Gibon et al. 2006).

Although high-throughput functional genomic methods, such as transcriptomics and metabolomics have identified key genes and metabolites involved in seed germination (Fait et al. 2006; Angelovici et al. 2011; Toubiana et al. 2012; Dekkers et al. 2013), there is a lack of integration of these studies, e.g. combining transcriptomics with metabolomics, to further zoom in on key metabolic pathways during successive developmental stages, including genes involved in C/N sources. Integration of *omics* data may constitute an alternative and complementary strategy to identify target genes and their ultimate products (metabolites) regulating either single biochemical pathways or more complex developmental mechanisms (Mercke et al. 2004). Powerful tools are now available for discovering links between transcripts and pathways of plant metabolism (Gutiérrez et al. 2008; Verdier et al. 2013; Cañas et al. 2015). High-throughput metabolomics approaches produce extensive metabolite data sets which can be combined with gene expression data (Fait et al. 2006). The combination of transcript and metabolite profiles have also been represented as networks, including both transcripts and metabolites (Holdsworth et al. 2008; Fukushima et al. 2011; Lv et al. 2014), which have been successfully used to discover regulatory and biosynthetic genes involved in the control of metabolite production (Verdier et al. 2013).

The present study highlights the dynamics of metabolism across developmental stages during the seed-to-seedling transition in Arabidopsis. Metabolite–metabolite correlation analysis underlined two profiles that seem to be implied in the metabolic preparation of seed germination and of efficient seedling establishment, respectively. In addition, we analysed combined transcriptomics and metabolomics data, which provides a general framework illustrating the significance of correlating metabolites and transcripts in the seed-to-seedling transition.

## Results

### Metabolic profiling of the seed-to-seedling transition

**Fig. 1:**
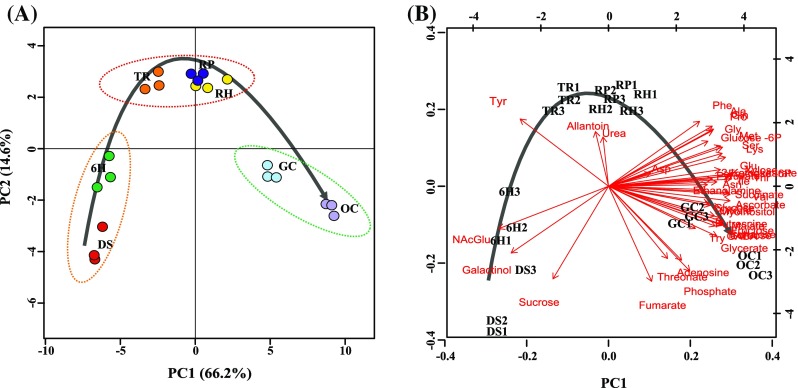
**a** Principal component analysis (PCA) of metabolite profiling at seven developmental stages across the seed-to-seedling transition. Data points of the same colour represent sample replicates. Principal component 1 (PC1) explained 66.2% of metabolite variance at developmental stages and principal component 2 (PC2) explained 14.6%. Dashed ellipses show three clusters: initial stages of germination (DS and 6H); early-seedling stages (TR, RP and RH) and last stages of seedling establishment (GC and OC). **b** Significant changes of the metabolites illustrated in a Biplot derived from the PCA-plot. Developmental stages are represented by: *DS* dry seed, *6H* six hours imbibed, *TR* testa rupture, *RP* radicle protrusion, *RH* root hair, *GC* greening cotyledons, and *OC* cotyledons fully opened. Arrow indicates the development

Upon GC-TOF-MS analysis of seven developmental stages of the seed-to-seedling transition (Fig. S1) we detected 183 metabolite peaks. From these, 43 could be identified and 140 are corresponding to unknown metabolites (Dataset S1 and Fig. S1). Identified and unknown metabolites were subjected to one-way ANOVA and differences in metabolite levels between developmental stages were considered significant if *p* < 0.01 (FDR adjusted). Forty-two identified and 124 unknown metabolites varied significantly between at least two developmental stages of the seed-to-seedling transition (Dataset S2). Principal component analysis (PCA) applied to the entire metabolome data set (identified and unknown compounds) showed an overall variation in metabolite levels among developmental stages (Fig. 1a). The high score (66.2%) for principal component one (PC1), which coincides with seed-to-seedling development, shows that there are considerable changes in metabolite composition during this transition. A biplot, displaying the major contribution of identified metabolites to the different developmental stages (Fig. 1b) indicates that dynamic changes in metabolite levels occur across the developmental stages. Galactinol, sucrose and N-acetylglutamic acid (NAcGlu) represented the most dominant metabolites in DS and 6H, whereas for the TR, RP and RH stages tyrosine, allantoin and urea were the most prominent metabolites. Most of the metabolites accumulated mainly in the final two stages of seedling establishment (GC and OC).

#### Overall changes in carbohydrates

**Fig. 2:**
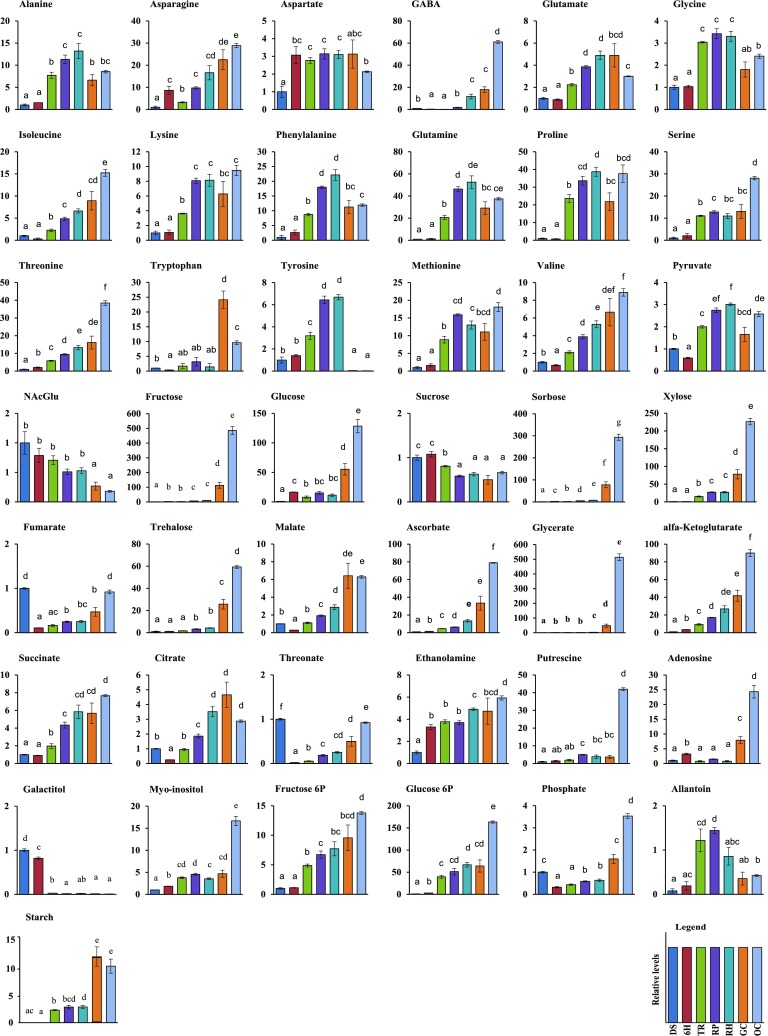
Relative concentration of 42 metabolites and starch which showed statistically significant variation during the seed-to-seedling transition. Different lower case letters above bars represent differences between samples by Tukey’s HSD (*p* < 0.05). Colours of bars refer to developmental stages. From left to right: *DS* dry seed, *6H* six hours imbibed, *TR* testa rupture, *RP* radicle protrusion, *RH* root hair, *GC* greening cotyledons, and *OC* cotyledons fully opened

In total ten carbohydrates were identified (fructose, glucose, sorbose, sucrose, trehalose, xylose), including two phosphor carbohydrates (fructose-6-phosphate and glucose-6-phosphate) and two sugar alcohol (galactinol and myoinositol). Levels of sorbose (twofold), fructose (threefold), glucose-6-phosphate (threefold) and glucose (16-fold) were enhanced during the first six hours of imbibition and kept increasing thereafter (Fig. 2), resulting in an increase in the levels of sucrose-derived monosaccharides at TR, such as fructose-6-phosphate (fourfold) and glucose-6-phosphate (13-fold). Only sucrose and galactinol showed a continuous decrease across the seed-to-seedling developmental stages. An initial reduction in sucrose content was observed at TR to about 40% of the content at 6H, suggesting that in the TR stage carbohydrate mobilization and metabolism are highly active (Fig. 2). In addition, sugars such as fructose, glucose, sorbose, xylose, trehalose, and glucose-6-phosphate increased during the final two stages of seedling establishment from twofold (GC) up to 10-fold (OC).

#### Overall changes in intermediates of the tricarboxylic acid cycle (TCA)

Nine organic acids were identified (ascorbate, citrate, fumarate, glycerate, malate, pyruvate, succinate, threonate and α-Ketoglutarate). Intermediates of the TCA-cycle, such as citrate, fumarate and malate showed levels 3- to 10-fold greater in DS than at 6H (Fig. 2). In contrast, levels of α-ketoglutarate increased threefold at 6H, as compared to DS. In addition, all identified TCA cycle intermediates showed a constant increase from TR to OC.

#### Overall changes in amino acid contents

Eighteen amino acids were identified, of which four changed in content during the first six hours of imbibition. An increase was detected for asparagine (eightfold), aspartate (threefold) and threonine (twofold) from DS to 6H, whereas pyruvate content at 6H was 50% lower than at DS (Fig. 2). However, most of the amino acids (15) increased at TR or later stages. Three of these 15 displayed a considerable boost in their levels at TR when compared to 6H; isoleucine (eightfold), glutamine (16-fold) and proline (32-fold) (Fig. 2). The shift from the last stage of germination (RP) to the first stage of seedling establishment (RH) was accompanied by an increase in gamma-aminobutyric acid (GABA) levels of around sixfold. When photosynthesis became active (GC), levels of tryptophan increased vastly to around 17-fold compared with RH. Three other amino acids did not display a strong increase during the RH to GC transition, but increased only around twofold (serine and threonine) and threefold (GABA) in OC compared with GC. Whereas all amino acids attained their highest levels at GC and OC stages, tyrosine was a notable exception as it was up to 149-fold higher at RP and RH when compared to GC and OC (Fig. 2).

### Metabolic correlation network analysis

**Fig. 3:**
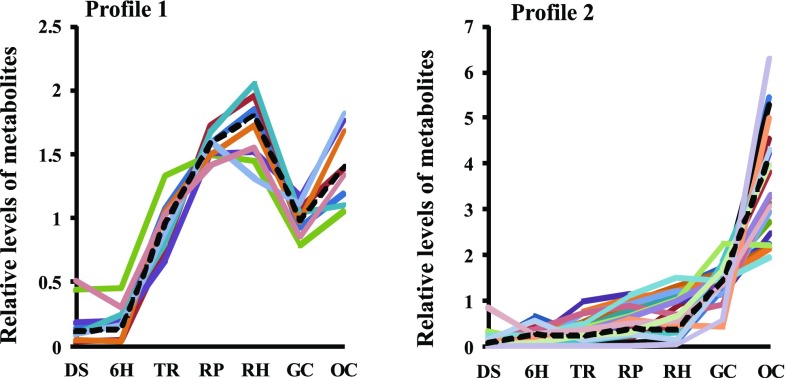
Two profiles of metabolite levels at developmental stages of the seed-to-seedling transition. Profiles were identified using *Fuzzy K-means* clustering. The black line depicts the relative median of metabolite levels in each profile. Developmental stages are represented by: *DS* dry seed, *6H* six hours imbibed, *TR* testa rupture, *RP* radicle protrusion, *RH* root hair, *GC* greening cotyledons, and *OC* cotyledons fully opened

We next explored the metabolic shifts between the heterotrophic and photoautotrophic states. We searched for groups of metabolites that display a similar concentration pattern across the different developmental stages by using the *Fuzzy K-Means* clustering method (Belostotsky et al. 2009) with some modifications (*Materials and Methods*). We identified two dominant metabolite profiles (Fig. 3).

Profile 1 included seven amino acids (alanine, glutamine, glycine, lysine, phenylalanine, proline, and methionine) and pyruvate, whereas Profile 2 was comprised of six amino acids (asparagine, GABA, isoleucine, serine, threonine, and valine), five carbohydrates (fructose, glucose, sorbose, xylose and trehalose), five organic acids (malate, α-ketoglutarate, succinate, ascorbate and glycerate), three carbohydrate derivatives (myo-inositol, fructose-6-phosphate and glucose-6-phosphate), adenosine, putrescine and phosphate (Fig. 3). Profile 1 represents metabolites that did not change much until 6H but increased steadily up to a maximum at the RH stage, followed by a decline to the GC stage and a subsequent increase to OC. Profile 2 represents metabolite levels that gradually increase across all the seed-to-seedling developmental stages and reached the highest level at OC. The three metabolites (sucrose, NAcGlu and galactinol) that were not included in these two dominant profiles displayed an opposite trend to Profile 2, that is they gradually decreased during development. The difference in numbers of metabolites between the patterns likely reflects variations in biochemical pathways during seedling development.

**Fig. 4:**
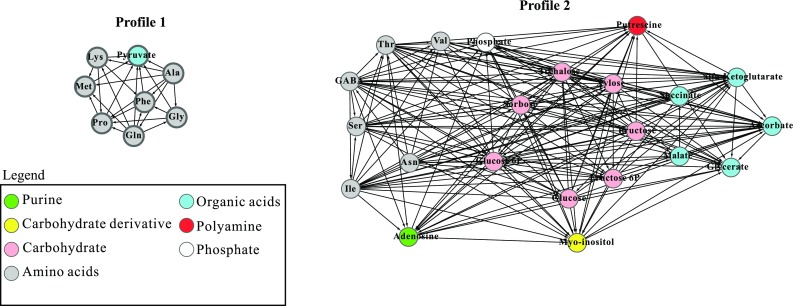
Metabolite profile networks, in which metabolites display higher connectivity to each other. Each edge in the networks indicates a source-target correlation

To characterize the profiles, we built a metabolite–metabolite correlation network for each profile (Fig. 4). Each edge of the networks represents a source-target (outgoing-incoming edges) correlation. A high number of incoming edges into a metabolite indicates the dependence of that metabolite on the outgoing metabolite edge (Xue et al. 2013).

Profile 1 shows an increase in levels of seven amino acids and of pyruvate at TR, representing key metabolites for an initial energy boost for seed germination. Profile 2 showed a high number of incoming edges for carbohydrates, organic acids, myo-inositol, putrescine and phosphate, whereas the amino acids showed a low number of incoming edges. Six amino acids present in Profile 2, besides a few incoming edges, showed high numbers of outgoing edges with asparagine showing outgoing edges only (Fig. 4). In Profile 2, metabolites such as carbohydrates and TCA cycle intermediates increased across all stages. Increased levels of metabolites in Profile 2, especially those of TCA cycle intermediates, may represent a cluster of metabolites required for successful seed germination, as well as seedling establishment.

To delineate the biochemical pathways of each profile, we performed biochemical pathway enrichment analysis using the MetaboAnalyst3.0 website tool (Dataset S4 and Table 1). Profile 1 was significantly (*p* < 0.01) enriched for biochemical pathways such as *aminoacyl-tRNA biosynthesis, nitrogen metabolism, alanine, asparagine and glutamate metabolism*, and *carbon fixation in photosynthetic organisms*. Profile 2 was enriched for *alanine, aspartate and glutamate metabolism, TCA cycle, aminoacyl-tRNA biosynthesis, galactose metabolism*, and *valine, leucine and isoleucine biosynthesis* (Table 1).

**Table 1:** Significantly enriched pathways for Profiles 1 and 2

Pathway name	Total	Hits	*p*	− log(*p*)	Holm *p*	FDR
Profile 1						
Aminoacyl-tRNA biosynthesis	67	7	6E − 09	2E + 01	5E − 07	5E − 07
Nitrogen metabolism	15	3	7E − 05	1E + 01	6E − 03	3E − 03
Alanine, aspartate and glutamate metabolism	22	3	2E − 04	8E + 00	2E − 02	7E − 03
Carbon fixation in photosynthetic organisms	21	2	7E − 03	5E + 00	6E − 01	1E − 01
Profile 2						
Alanine, aspartate and glutamate metabolism	22	4	4E − 04	8E + 00	3E − 02	3E − 02
Citrate cycle (TCA cycle)	20	3	4E − 03	5E + 00	4E − 01	1E − 01
Aminoacyl-tRNA biosynthesis	67	5	4E − 03	5E + 00	4E − 01	1E − 01
Galactose metabolism	26	3	9E − 03	5E + 00	7E − 01	2E − 01
Valine, leucine and isoleucine biosynthesis	26	3	9E − 03	5E + 00	7E − 01	2E − 01

Summary of the significant metabolite pathways in each profile ranked by their *P* values (*p* < 0.01). Total is the total number of compounds in the pathway; Hits is the actually matched number from each profile; Holm *p* is the *p* value adjusted by Holm-Bonferroni method and FDR is the *p* value adjusted using the false discovery rate adjustment method

### Comprehensive metabolic pathways

**Fig. 5:**
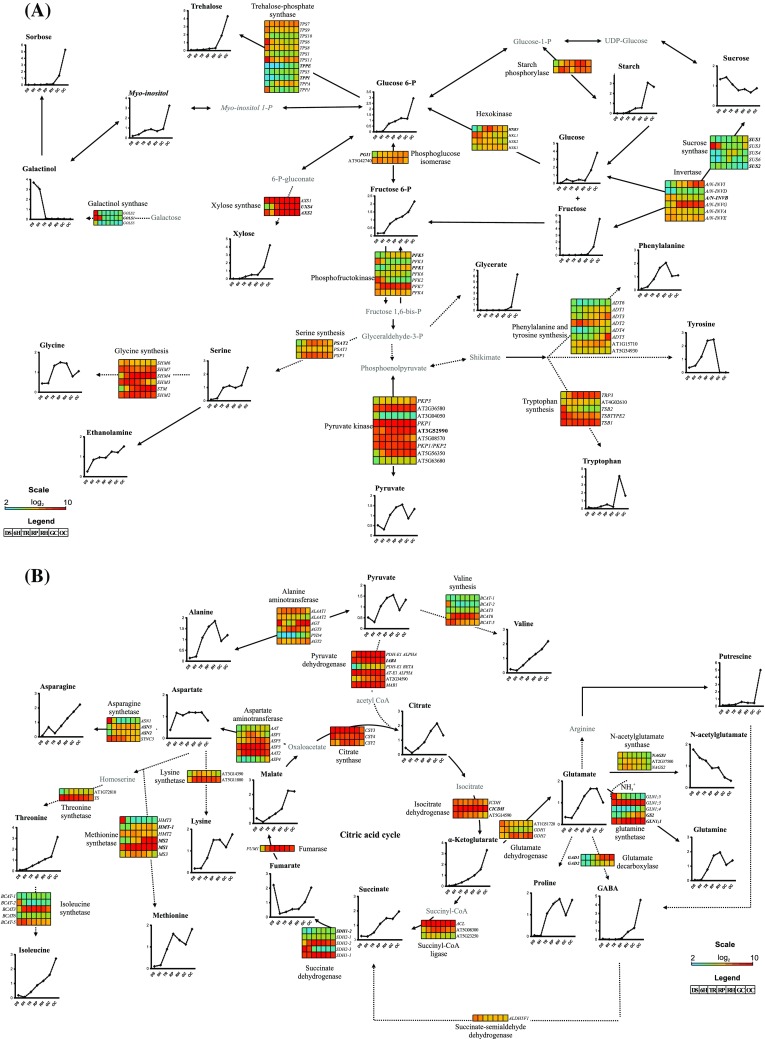
Comprehensive primary metabolic map of the seed-to-seedling transition in Arabidopsis for **a** carbohydrate, **b** TCA and **a**, **b** amino acid metabolism. On the metabolic map 147 transcripts from Silva et al. (2016) and 36 metabolites are mapped to three main metabolic groups of compounds: carbohydrates, organic acids and amino acids. Metabolite data are means of three replicates using 350 seeds for each replicate. According to previous annotations, expression profiles of transcripts encoding enzymes involved in the metabolite productions are represented. Genes indicated in boldface are highly correlated (*r* > 0.85) to their respective metabolites

In a previous study, we obtained transcriptome data from the seven studied developmental stages of the seed-to-seedling transition [Silva et al. (2016)]. This transcriptome dataset provides a comprehensive description of gene expression during the seed-to-seedling transition. We mapped 147 representative transcripts, which are associated with energy and amino acid metabolism at the seven stages (Fig. 5), focusing on the changes in gene expression and metabolite levels associated with carbohydrate, organic acid, and amino acid metabolism. Displaying the transcripts and metabolites in this way revealed that changes in transcript abundance preluded the later changes in metabolite contents. Importantly, 42 out of 147 transcripts were involved in the metabolism of eight carbohydrates and their derivatives (sucrose, glucose, fructose, fructose 6-phosphate, glucose 6-phosphate, trehalose, xylose, myo-inositol and galactinol), whereas 34 were involved in the metabolism of six organic acids (pyruvate, citrate, α-ketoglutarate, succinate, fumarate and malate). Furthermore, metabolic pathways involving 71 transcripts were associated with 17 amino acids (glycine, serine, phenylalanine, tyrosine, tryptophan, alanine, valine, asparagine, aspartate, lysine, threonine, isoleucine, methionine, glutamate, GABA, glutamine and NAcGlu). Fifteen transcripts associated with carbohydrate metabolism were positively correlated with the corresponding metabolite levels (Fig. 5a): sucrose levels with transcript abundance of *INVERTASE B* (*A*/*N-INVB*), which encodes an enzyme involved in the hydrolysis of sucrose; expression of *SUCROSE SYNTHASE 1* (*SUS1*) and *SUCROSE SYNTHASE 2* (*SUS2*) with fructose and sucrose contents, respectively. Sucrose synthase catalyzes the conversion of glucose and fructose to sucrose and vice versa. Glucose is also associated with a biochemical reaction involving hexokinase, in which hexokinase phosphorylates glucose to glucose-6-phosphate. The expression of two isoforms of hexokinase, *HEXOKINASE 1* (*HXK1*) and *HEXOKINASE 3* (*HXK3*) correlated well with glucose-6-phosphate levels. Glucose-6-phosphate, besides the above biochemical reactions, is also involved in an important step of glycolysis: its conversion to fructose-6-phosphate. This conversion is mediated by phosphoglucose isomerase and, indeed, *PHOSPHOGLUCOSE ISOMERASE 1* (*PGI1*) expression correlated with glucose-6-phosphate content. Another important biochemical reaction that incorporates fructose-6-phosphate into glycolysis involves fructokinases which indirectly convert fructose to fructose-6-phosphate. The expression of *PHOSPHOFRUCTOKINASE 1* (*PFK1*) and *PHOSPHOFRUCTOKINASE 5* (*PFK5*) was associated with fructose content. Glucose-6-phosphate is also a precursor for the biosynthesis of trehalose. The expression of four genes encoding trehalose-phosphate synthases correlated well with trehalose and glucose-6-phosphate; the expression of *TREHALOSE-6-PHOSPHATE PHOSPHATASE E* (*TPPE*) and *TREHALOSE-6-PHOSPHATE PHOSPHATASE I* (*TPPI*) had the highest correlation with trehalose. Glucose-6-phosphate, however, was linked with *TREHALOSE-6-PHOSPHATASE SYNTHASE S5* (*TPS5*) and *TREHALOSE-6-PHOSPHATE PHOSPHATASE A* (*TPPA*) transcript levels. Xylose and galactinol levels were in agreement with transcript levels of enzymes associated with their biosynthesis: *UDP-D-XYLOSE SYNTHASE 2* (*AXS2*) and *UDP-XYLOSE SYNTHASE 4* (*UXS4*), and *GALACTINOL SYNTHASE 1* (*GOLS1*) expression, respectively. The number of transcripts correlating with organic acid metabolism was lower than for carbohydrate metabolism. Four transcripts correlated positively with the three organic acids, pyruvate, succinate and α-ketoglutarate. Pyruvate is involved in two important pathways, glycolysis and the TCA cycle. Levels of pyruvate were clearly associated with the expression of *PYRUVATE KINASE* (At3g52990) (Fig. 5a) and *PYRUVATE DEHYDROGENASE E1A-LIKE SUBUNIT* (*IAR4*) (Fig. 5b). Pyruvate kinase transfers the phosphate group from phosphoenolpyruvate (PEP) to ADP, resulting in pyruvate, after which pyruvate dehydrogenase transforms pyruvate into acetyl-CoA, which is incorporated into the TCA cycle. The expression of the gene encoding isocitrate dehydrogenase (IDH) correlated with α-ketoglutarate levels. This enzyme catalyzes the conversion of isocitrate into α-ketoglutarate. The expression of *CYTOSOLIC NADP* ^+^ *-DEPENDENT ISOCITRATE DEHYDROGENASE* (*CICDH*) correlated with α-ketoglutarate levels and succinate with transcript levels of one isoform of succinate dehydrogenase (*SUCCINATE DEHYDROGENASE 1-2* (*SDH1-2*), which is an enzyme that catalyses the conversion of succinate to fumarate. It seems to be the isoform that catalyses the conversion of isocitrate to α-ketoglutarate (Fig. 5b).

Abundance of 13 transcripts associated with amino acid metabolism was positively correlated with levels of eight amino acids (Fig. 5b). Aspartate is a precursor of asparagine through asparagine synthetase. We examined the correlation of isoforms of this enzyme, but none of the transcript levels correlated with aspartate; however, *ASPARAGINE SYNTHETASE 2* (*ASN2*) and *ASPARAGINE SYNTHETASE 3* (*ASN3*) transcript levels did correlate with asparagine. Another amino acid, methionine, was found to correlate very well with the transcript levels of three isoforms of methionine synthetase, *METHIONINE SYNTHASE 1* (*MS1*), *METHIONINE SYNTHASE 2* (*MS2*) and *HOMOCYSTEINE S-METHYLTRANSFERASE* (*HMT-1*). Glutamate decarboxylase (GAD) is the enzyme responsible for decarboxylation of glutamate to GABA. The expression of two *GAD* isoforms identified during the seed-to-seedling transition was well associated with glutamate and GABA content. *GLUTAMATE DECARBOXYLASE 1* (*GAD1*) correlated with glutamate and GABA, whereas *GLUTAMATE DECARBOXYLASE 2* (*GAD2*) only showed correlation with glutamate. Glutamate is converted to N-acetylglutamate, a reaction catalysed by N-acetylglutamate synthase; however, glutamate was not appreciably associated with isoforms of this enzyme. *N*-acetylglutamate levels correlated with *N-ACETYL-L-GLUTAMATE SYNTHASE 1* (*NAGS1*) (Fig. 5b). Glutamate is also precursor in the formation of glutamine, a reaction catalysed by glutamine synthetase. Glutamate, as well as glutamine, showed very good correlations with transcript levels of two isoforms of glutamine synthetase, *GLUTAMINE SYNTHASE 1;1* (*GLN1;1*) and *GLUTAMINE SYNTHETASE 2* (*GS2*), suggesting that these two isoforms play a role in the conversion of glutamate to glutamine during germination and early seedling establishment. Phenylalanine correlated well with *AROGENATE DEHYDRATASE 1* (*ADT1*), which is involved in phenylalanine biosynthesis (Fig. 5a). Of the three isoforms of genes involved in serine biosynthesis only the expression of *PHOSPHOSERINE AMINOTRANSFERASE 2* (*PSAT2*) correlated with the serine level (Fig. 5a). Taken together, each correlation between gene expression and metabolite content involved in the metabolism of primary metabolites highlights a potential role for these genes and their products in the seed-to-seedling transition in Arabidopsis.

## Discussion

This study focused on metabolic changes during the seed-to-seedling transition in *Arabidopsis thaliana* in order to determine how the advancement of growth is associated with certain metabolite profiles. This transition involves several developmental stages ranging from DS (mature dry seed) to OC (opening of cotyledons, i.e. the last stage of early seedling establishment). In this study we performed a detailed profiling of primary metabolites, using morphological markers of development.

Radicle protrusion (RP) is considered the morphological marker defining the transition between completion of germination and commencement of seedling development (Bewley et al. 2013). Three stages prior to RP and three stages thereafter were selected for metabolite profiling. A previous study employing metabolite profiling in developing seedlings was targeted at heterosis of two Arabidopsis genotypes (Col-0 and C24) (Meyer et al. 2012). Moreover, in that study samples were selected based on temporal markers rather than morphological markers. For example, samples collected 48 h after imbibition were analysed, but these samples consisted of a mixture of three developmental stages: TR, RP, and RH because of biological variation in the germination speed of individual seeds of the population (Meyer et al. 2012). Despite the fact that metabolite profiling has been studied in seed germination before (Fait et al. 2006; Shu et al. 2008; Angelovici et al. 2011; Joosen et al. 2013), many questions regarding the regulation of the seed-to-seedling transition remain open, due to the lack of a comprehensive analysis of changes in metabolism by combining expression analysis and metabolite levels. The metabolite profiles of the seven seed-to-seedling developmental stages displayed significant variation among them, not only in metabolite content but also in metabolite–metabolite network correlations. In combination with transcriptomics data, we constructed a metabolic map. Thus, this study presents a broader and more precise metabolic analysis as compared to previous studies (Fait et al. 2006; Meyer et al. 2012).

Metabolic changes during the seed-to-seedling transition were first visualized in a PCA plot. This resulted in the observation of major shifts in metabolite profiles between three phases of this transition: (1) initial stages of germination (DS and 6 h); (2) stages of early seedling growth (TR, RP and RH), and (3) the final stages of seedling development (GC and OC) in which the seedling becomes photoautotrophic. Metabolic shifts are represented by the increasing distance between these different phases, i.e. galactinol, sucrose and NAcGlu, show markedly higher levels at DS and 6H, which, thus, separates them from other stages. In the early-seedling phase tyrosine, allantoin and urea are prominent metabolites, suggesting a role at this phase. The most significant metabolites that contribute to separation of the final stage are amino acids.

The detected carbohydrates and their derivatives such as glucose, fructose, sorbose, xylose, trehalose, fructose-6-phosphate and glucose-6-phosphate displayed high levels at OC, which implies that the carbon status was relatively high at the seedling stage, as compared to the previous stages. Additionally, amino acids with high contents at the GC and OC stages, such as glutamate, glutamine, asparagine and aspartate were variable among the developmental stages. It indicates a high variability of nitrogen status, despite the fact that these amino acids are known to be important for monitoring the C/N balance in plants (Zheng 2009). Since amino acids are important forms of N storage for seeds (Lohaus and Moellers 2000; Sanders et al. 2009), our results suggest that, at the metabolomic level, N status in amino acids represented by Profile 1 plays an important role in balancing C and N in the transition of the initial phase of germination to the early-seedling stage. For example, CO_2_ is assimilated through photosynthesis (at seedling stages) and, through conversion of sucrose and glucose in glycolysis and the tricarboxylic acid cycle (TCA) to α-ketoglutarate. This metabolite serves as a C skeleton for the synthesis of glutamate by incorporating photorespiratory NH_4_^+^, which results in the production of glutamate and glutamine to donate NH_4_^+^ for the synthesis of all other amino acids (Zheng 2009) to fuel primary metabolism. Taken together, it explains the high abundance of metabolites such as glucose, α-ketoglutarate and amino acids at seedling stages. Furthermore, amino acids show outgoing edges to pyruvate within the metabolite–metabolite network. It is known that aminotransferase performs a reversible reaction converting these amino acids to pyruvate and vice versa (Orzechowski et al. 1999; Pinto et al. 2014; McAllister and Good 2015). Additionally, amino acids present in Profile 1 participate substantially in nitrogen transport (Orzechowski et al. 1999; Yoo et al. 2013; Pinto et al. 2014; McAllister and Good 2015).

Different from Profile 1, Profile 2 shows a constant increase in metabolites with highest levels at the final stages of seedling establishment. This pattern represents the metabolic shift from the early-seedling stage to the last phase of seedling development. Furthermore, the metabolic network of Profile 2 shows a high number of incoming edges for glycolytic and TCA intermediates, which confirms the strong relationship between regulation of glycolysis and the TCA cycle. Amino acids present in Profile 2, such as GABA, isoleucine, aspartate, serine, threonine and valine may also influence the C/N balance. For example, GABA is associated with succinate in the TCA cycle, which may provide high levels of succinate during the seed-to-seedling transition. This could be related to the established role of GABA in regulating the C/N ratio when carbon supply is limited (Michaeli et al. 2011). Besides carbohydrates, amino acids and organic acids, starch also displayed a steady increase across the seed-to-seedling transition. This result corroborates a previous study in Arabidopsis (Matsoukas et al. 2013) in which starch metabolism was associated with the juvenile-to-adult plant phase transition during normal growth and development. It was suggested that plants in the juvenile phase may require starch accumulation to reach a threshold level in order to sustain a steady supply of maltose and/or sucrose during the juvenile-to-adult phase transition (Matsoukas et al. 2013). This observation may, thus, extend to the seed-to-seedling phase transition, where there is an opposite trend of changes in amino acid-, sucrose- and galactinol contents, suggesting a shift from carbon- to nitrogen metabolism. These marked changes in sugar levels are predominantly associated with major carbohydrate metabolism. Although sucrose levels decreased, the abundance of all other carbohydrates, including starch, increased, which suggests a substantial rate of import of the products from reserve mobilization into glycolysis and that starch may be produced from the sucrose source.

Profile 1 and 2 share two enriched pathways (*aminoacyl-tRNA biosynthesis* and *alanine, asparagine and glutamate metabolism*). It is known that aminoacyl-tRNAs are present in the dry seed to permit immediate resumption of protein synthesis in the seed upon imbibition (Desai et al. 1997). It suggests that the seven amino acids of profile 1 (phenylalanine, glutamine, glycine, methionine, alanine, lysine and proline) play an important role in the resumption of protein synthesis for seed germination, whereas the five amino acids of profile 2 (asparagine, serine, valine, isoleucine and threonine) are involved in the continuation of seedling development and -establishment. Moreover, the enrichment of *alanine, asparagine and glutamate metabolism* implies that the opposite occurs with more metabolites in Profile 2 (asparagine, GABA, alfa-ketoglutarate and succinate) than in Profile 1 (alanine, glutamate and pyruvate). It should also be noted that glutamate is the precursor for chlorophyll synthesis in developing leaves (Yaronskaya et al. 2006). Taken together, our results suggest that the metabolites in Profile 1 are important for the first exposure of cotyledons to light and the metabolites of Profile 2 in the preparation for development of the first true leaves.

These profiles also reflect the timing of metabolite changes, principally in profile 1, in which metabolites show a transient increase from the RP to the RH stage. Methionine a fundamental metabolite in its function of building blocks for protein synthesis (Ravanel et al. 1998), is present in profile 1. Methionine is an important precursor of glucosinolates which are associated with pathways that control plant growth and development (Grubb and Abel 2006). RNAi-inhibited expression of *CYTOCHROME P450 79F1*(*CYP79F1*) and *CYP79F2* in Arabidopsis resulted in a significant accumulation of methionine during plant development (Chen et al. 2012). This is because *CYP79F1* and *CYP79F2* catalyze the biosynthesis of short-chain and long-chain aliphatic glucosinolates using chain-elongated methionine substrates (Grubb and Abel 2006). Another study with a *cyp79f1* mutant, and co-suppression of *CYP79F1* and *CYP79F2* yielded plants with strong phenotypes such as dwarf, bushy, and semi-sterile (Reintanz et al. 2001). Targeting different pathways, a study using a proteomic approach showed the importance of methionine for seed germination and, consequently, for seedling development (Gallardo et al. 2002). These authors demonstrated that methionine synthase increased strongly during the first 24 h after imbibition (HAI), prior to radicle protrusion, but the level of this enzyme did not increase further at 48 HAI, which coincided with radicle protrusion. However, another enzyme (S-adenosylmethionine (AdoMet)) accumulated at the radicle protrusion stage. Consistent with an important role of methionine during the seed-to-seedling transition, a specific inhibitor of methionine (DL-propargylglycine) delayed seed germination and blocked seedling establishment; however, the phenotypes were recuperated after transfer to a medium supplemented with methionine (Gallardo et al. 2002). In conclusion, methionine is an important amino acid for seedling development, likely as a key precursor of glucosinolate pathways, influencing in the final developmental stages (GC and OC).

In addition to providing potential targets for the engineering of seed-to-seedling transition metabolism, this study also allowed a general assessment of transcriptional regulation during this transition. Previous studies have suggested that regulation of metabolism occurs at the post-translational level (Jiao and Chollet 1991; Kolbe et al. 2006; Lea et al. 2006; Bates et al. 2014; Pisithkul et al. 2015). However, we identified several linear correlations between gene expression and metabolite levels. For example, strong correlations of carbohydrates and their derivatives with transcripts associated with glycolysis, suggest that metabolism is also regulated transcriptionally. For example, sucrose levels correlated well with transcript levels of sucrose synthase (*SUCROSE SYNTHASE* 2 - *SUS2*) and invertase (*INVERTASE B* - *INVB*). Several studies have shown the importance of these enzymes for normal development (Cheng et al. 1996; Yau and Simon 2003; Barratt et al. 2009). Our results suggest that INVB and SUS2 may be the key enzymes essential for sucrose metabolism in support of normal seedling establishment. This corroborates previous reports which demonstrated an important role for *INV1* in root cell development and reproduction in rice (Jia et al. 2008) and for whole plant development in *Lotus japonicus* (Welham et al. 2009). It is not clear whether INVB and SUS2 have regulatory properties that allow flux of C out of sucrose via this pathway in coordination with energy demands by the cells, or whether *SUCROSE SYNTHASE1* (*SUS1*), which is well correlated with fructose content, could compensate for the lack of sucrose to maintain fuelling of energy-demanding processes. Transcript abundance of two isoforms of hexokinase (*HEXOKINASE 1* - *HXK1* and *HEXOKINASE 3* - *HXK3*) correlated well with glucose-6-phosphate levels. Hexokinase is an enzyme in the glycolysis pathway and its main substrate is glucose, which also has a function in the control of plant development and expression of different classes of genes (Renz and Stitt 1993; Dai et al. 1999; Claeyssen and Rivoal 2007). A previous study has shown that the C flux through hexokinase activity exhibits a high control over glucose during normal root growth (Claeyssen et al. 2013). This suggests that the *HXK1* and *HXK3* encoded enzymes control a step in glycolysis during the seed-to-seedling transition. Besides the hexokinase reaction (phosphorylation of glucose to form glucose-6-phospate), glucose-6-phosphate is precursor for the formation of fructose-6-phosphate through phosphoglucose isomerase (*PGI*). *PGI1* transcript abundance was well correlated with glucose-6-phosphate levels. It has been shown that *PGI1* plays a role in the transition to flowering in Arabidopsis. The *pgi1-1 mutant* flowers earlier than wild type (Yu et al. 2000). However, the phenotype could be reverted to the wild type by the addition of sugars (glucose, fructose and sucrose), suggesting that it is an important enzyme for a developmental phase transition. Our results show that *PGI1* may be a key enzyme for the supply of C to glycolysis through glucose-6-phosphate during the seed-to-seedling transition.

Organic acid levels also showed good correlations with transcripts associated with their biosynthesis. The last step of glycolysis is the conversion of phosphoenolpyruvate to pyruvate via pyruvate kinase (PKP) towards the TCA cycle. Expression of a putative pyruvate kinase (At3g52990) correlated well with pyruvate levels. Several studies have shown the importance of PKP in seed germination (Baud et al. 2007) and in seedling establishment (Andre and Benning 2007) in Arabidopsis. These studies have shown that *pkp* mutants exhibit delayed germination (Baud et al. 2007) and that PKP plays an important role in catabolizing storage compounds in germinating Arabidopsis seeds (Andre and Benning 2007). The correlation of At3g52990 transcript abundance with pyruvate levels, suggests that also this putative pyruvate kinase isoform plays a role during seedling establishment, although this gene has not been described before in this particular context. Pyruvate is also related to another key reaction via pyruvate dehydrogenase, an enzyme that catalyzes the oxidative decarboxylation of pyruvate, yielding CO_2_, acetyl-CoA and NADH (Reed 1974). Interestingly, our results show that pyruvate levels correlate well with the transcript abundance of the pyruvate dehydrogenase *E1a-lik*e (*IAR4*), which is 81% identical to a previously characterized Arabidopsis mitochondrial PDH E1α-subunit (*AT-E1 ALPHA -* At1g59900) (Quint et al. 2009). However, *AT-E1 ALPHA* transcript abundance showed a negative correlation with pyruvate levels. The importance of pyruvate dehydrogenase has been demonstrated clearly by a mutation in the E2 subunit, which reduced plant organ size and increased accumulation of pyruvate (Yu et al. 2012). Our results suggest that *IAR4* is the gene controlling the entry of C, through acetyl-CoA, into the TCA cycle for energy production during the seed-to-seedling transition. In the TCA cycle, *CYTOSOLIC NADP*+*-DEPENDENT ISOCITRATE DEHYDROGENASE* (*CICDH*) transcript abundance is well correlated with citrate levels and this corroborates the observation that citrate accumulated in three independent knock-out mutants (*icdh-1, icdh-2*, and *icdh-*3) of Arabidopsis under normal growth conditions (Mhamdi et al. 2010).

Our results also show that amino acids such as aspartate, methionine, glutamate, NAcGlu, glutamine, phenylalanine, and serine correlated well and positively with transcripts related to their biosynthesis. Aspartate, glutamate and glutamine are involved in transamination processes and N assimilation (Zheng 2009). These amino acids show a continuous increase in content during the seed-to-seedling transition and correlated with transcripts associated with their biosynthesis, such as asparagine synthetase (*ASN2* and *ASN3*), glutamate decarboxylase (*GAD1* and *GAD2*) and glutamine synthetase (*GLN1;1* and *GS2*), which suggests that there is N assimilation. N assimilation may lead to the synthesis of other amino acids, such as lysine, phenylalanine, valine and methionine at the final stages of seedling establishment, when growth rate is relatively high.

**Fig. 6:**
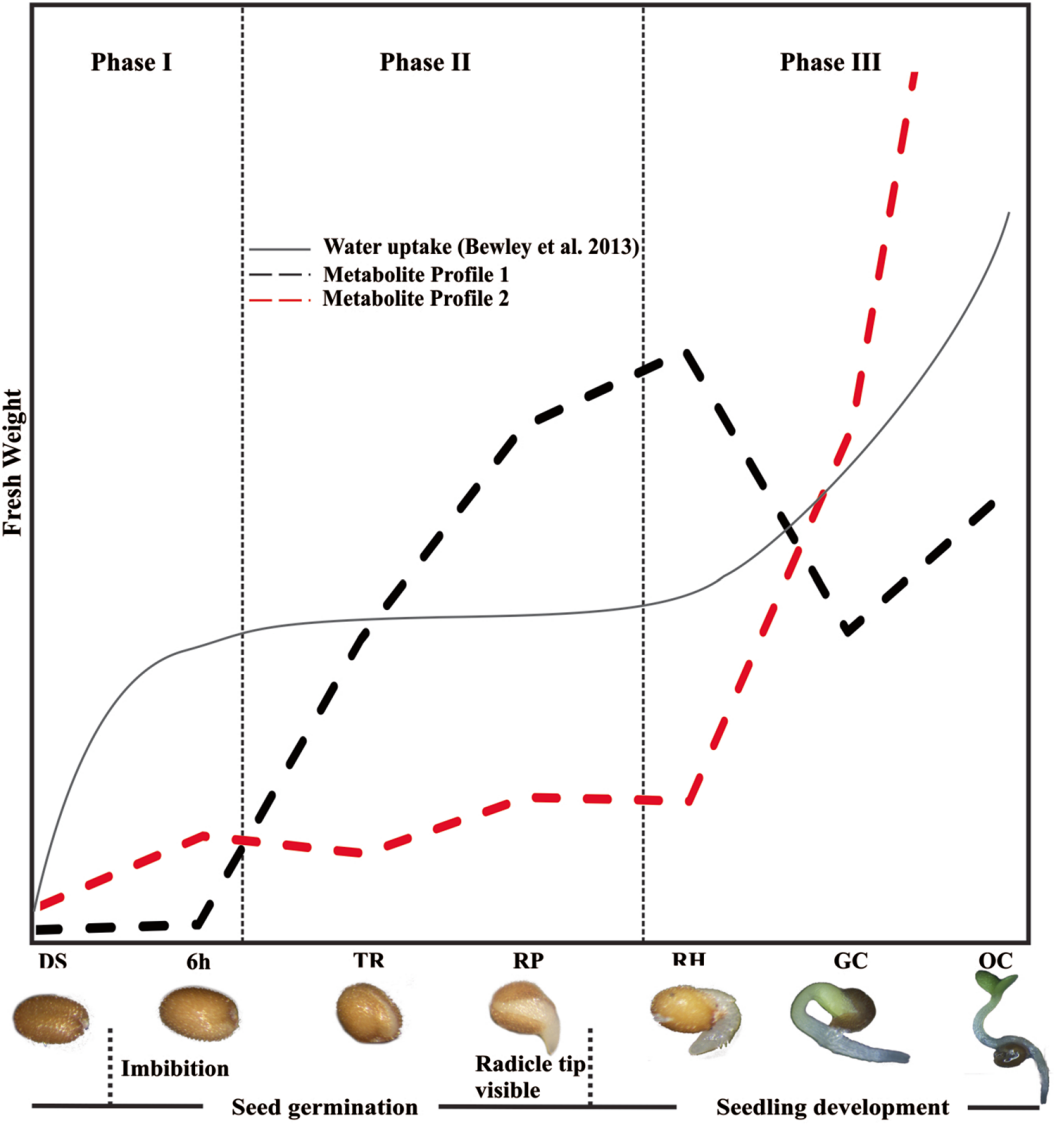
Metabolic shifts during the seed-to-seedling transition in seven developmental stages of Arabidopsis (Silva et al. 2016). The transition from Phase I to Phase II is characterized by levels of metabolites present in profile (1) Immediately preceding Phase III, seedling establishment is prepared with a boost of metabolites present in profile (2) Dashed lines distinguish metabolite profile 1 (black) and metabolite profile 2 (red)

Taken together, our results enable us to draw important conclusions concerning metabolism during the seed-to-seedling transition in Arabidopsis. We demonstrate that primary metabolism is co-ordinately regulated during the seed-to-seedling transition, displaying two major shifts that separate three groups of developmental stages (Fig. 6). Also, we show that the one to one correlation of metabolite content with gene expression during early seed development is quite considerable and hints at a predominantly transcriptional regulation of this stage of plant development. The first metabolic shift from the initial stages of germination (DS and 6H) to the early-seedling stages (TR, RP and RH) is represented by Profile 1, in which seven amino acids and pyruvate are present. The second metabolic shift occurs between early-seedling and the last stages of seedling establishment and is reflected by Profile 2 in which amino acids, organic acids, carbohydrates and their derivatives are present. These metabolic profiles together with the network analysis corroborate results by Allen et al. (2010), which indicated a shift in the state of nutritionally important metabolites that precedes the major shift in the transcriptional state, proceeding from germination to seedling emergence. Although transcriptional changes did not always equate to a proportional metabolic response, detailed correlation analysis enabled us to identify genes that do seem to directly influence the flux through metabolic pathways during the seed-to-seedling transition. Our study provides a detailed overview of the transcriptional and metabolic changes during the seed-to-seedling transition in Arabidopsis and opens new perspectives for understanding the complex regulatory mechanisms underlying this transition.

## Materials and methods

### Plant material

Seeds of *Arabidopsis thaliana*, accession Columbia (Col-0 [N60000]), were cold stratified at 4 °C in the dark for 72 h in Petri dishes on two layers of moistened blue filter paper (Anchor Paper Company, St Paul, MN, USA). Germination was performed in standard plant growth chambers at 22 °C under constant white light (12 Philips 36W Fluorescence 2084 tubes; 140 Wm^−2^). To elucidate the changes in metabolomes that prepare for and accompany the transition from a seed into a photoautotrophic seedling, three biological replicates for seven developmental stages during this transition were used for metabolite extraction as described below. The successive developmental stages were selected by Silva et al. (2016): (DS) mature dry seed; (6H) seed six hours upon imbibition; embryo swelling and (TR) testa rupture; (RP) protrusion of the radicle through the endosperm, followed by embryonic root growth and (RH) root hair formation, followed by (GC) greening cotyledons and (OC) fully opened cotyledons. We precisely selected individually 350 seeds and seedlings at seven well-defined developmental stages, rather than using temporal markers, to avoid variation in rate of development of the single seeds (Fig. S1).

### Metabolite extraction, derivatization and GC-TOF-MS analysis

Polar metabolites were extracted, derivatized and run on the GC-TOF-MS as described before (Ribeiro et al. 2014) but 5 mg of dry material was used instead of 20 mg. All volumes were stoichiometrically adjusted.

In order to confirm the sucrose trend from GC-TOF-MS, soluble carbohydrates were also determined as described previously (Ribeiro et al. 2014). The supernatant after starch extraction was injected into a Dionex HPLC system (ICS 5000 ^+^ DC) to analyse the soluble carbohydrate content, using a CarboPac PA 1, 4- × 250-mm column preceded by a guard column (CarboPac PA 1, 4 × 50 mm), and a gradient pump module (ICS 5000 Dual Pump, Dionex). Mono-, di-, and tri-saccharides were separated by elution in an increasing concentration of NaOH (20–350 mM) with a flow rate of 1 mL min^−1^. Peaks were identified by co-elution of soluble carbohydrate standards. Sugar quantity was corrected by mean of the internal standard (melezitose) and transformed to micrograms of sugar per milligram of dry material.

#### Data processing

Raw data was processed by ChromaTOF software 2.0 (Leco^®^), and further baseline correction, accurate mass calculation, data smoothing and noise reduction, followed by alignment between chromatograms were performed using the MetAlign software (Lommen 2009). MSClust was used to remove metabolite signal redundancy in aligned mass peaks tables and to retrieve mass spectral information of metabolites using mass peak clustering (Tikunov et al. 2012). The mass spectra of the representative masses were used for tentative identification by matching to the spectral libraries (National Institute of Standards and Technology [NIST08]; Golm metabolome database [http://gmd.mpimp-golm.mpg.de/]) and by comparison of the retention index calculated using a series of alkanes. Authentic reference standards were used to confirm the identity of the metabolites. Levels of identification are presented in Supplementary Dataset S1 according to Sumner et al. (2007).

#### Metabolomic analysis

The processed data was uploaded into MetaboAnalyst software (http://www.metaboanalyst.ca) according to the user’s guide (Xia et al. 2009; Xia and Wishart 2011). Data normalization was performed by adjustment of the concentrations based on biological input (dry weight) and reference feature (ribitol). Subsequently, generalized-logarithm transformation was performed, followed by unit scaling (mean-centred and divided by standard deviation of each variable). Multivariate analysis was performed using log transformed and scaled data. Statistically significant differences for the variables between seed-to-seedling transition developmental stages were tested by ANOVA followed by *post hoc* analysis for comparisons in multiple groups. The *p*-value resulting from ANOVA was adjusted to the false discovery rate-adjusted *p*-value (FDR). Principal component analysis (PCA) was performed on the entire data set.

#### Dominant profile, network and metabolite pathway enrichment analysis

*Fuzzy K-Means* clustering was used for identification of common patterns (Belostotsky et al. 2009). Pearson correlation was performed for each identified profile. A table for each profile with correlation coefficient values among metabolites was exported to Cytoscape V.2.8.2 (Smoot et al. 2011). Pathway enrichment analysis was performed for each profile using Metaboanalyst 3.0 (Xia et al. 2015).

### Transcript-metabolite correlations

To understand the regulation of the biochemical pathways that operate during the seed-to-seedling transition, we provide Pearson correlations between metabolite content and transcript abundance. Hereto, we used the transcriptome data of the same seven successive developmental stages as described previously (Silva et al. 2016). Dataset S3 presents the Pearson correlations between the levels of identified metabolites and transcripts of the corresponding metabolic pathway enzymes.

## Electronic supplementary material

Below is the link to the electronic supplementary material.

**Dataset S1**: Averaged raw metabolite abundance data with standard errors for the 43 identified metabolites. Data means of three biological replicates containing 350-400 seeds each are shown. (XLSX 52 KB)

**Dataset S2**: ANOVA post-hoc statistical results. Column shows the comparisons between different developmental stages that are significant given the *p* < 0.05. (XLSX 21 KB)

**Dataset S3**: Transcript-metabolite correlations. Columns show the Pearson correlation value for each comparison. (XLSX 43 KB)

**Dataset S4**: Assignment of metabolites to the profiles and their pathway enrichment. (XLSX 19 KB)

**Figure S1**: Sub-division of the seed-to-seedling developmental stages. DS – dry seeds; 6H – six hours imbibed; TR – testa rupture; RP – radicle protrusion; RH – root hair; GC – greening cotyledons and OC – cotyledons fully opened (Silva et al. 2016). (JPG 802 KB)
