# Supplementary figures and images for: Metabolite profiling and associated gene expression reveal two metabolic shifts during the seed-to-seedling transition in Arabidopsis thaliana
Source: Plant Mol Biol. 2017 Oct 18;95(4):481–96. doi: 10.1007/s11103-017-0665-x (PMC5688192; doi:10.1007/s11103-017-0665-x)

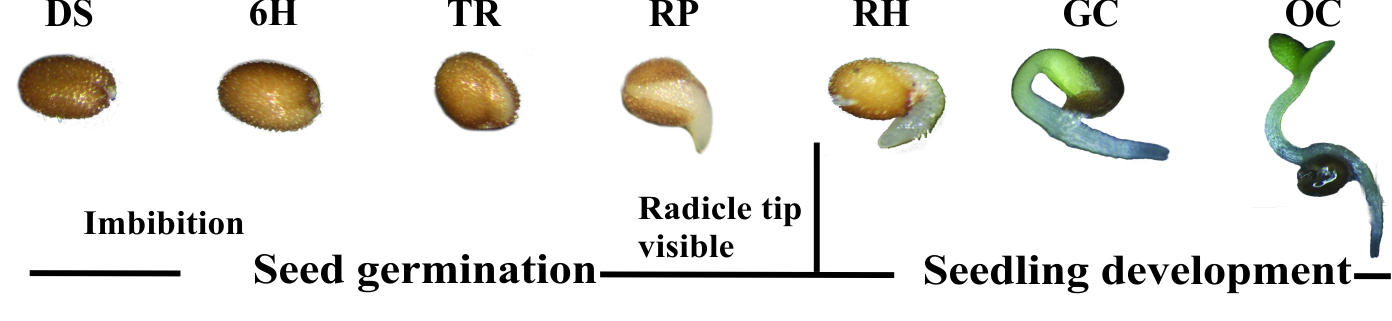

Supplement: Supplementary file 5 — Figure S1: Sub-division of the seed-to-seedling developmental stages. DS – dry seeds; 6H – six hours imbibed; TR – testa rupture; RP – radicle protrusion; RH – root hair; GC – greening cotyledons and OC – cotyledons fully opened (Silva et al. 2016). (JPG 802 KB) [file 11103_2017_665_MOESM5_ESM.jpg]
